# Utility Values for Advanced Soft Tissue Sarcoma Health States from the General Public in the United Kingdom

**DOI:** 10.1155/2013/863056

**Published:** 2013-03-17

**Authors:** Julian F. Guest, Erikas Sladkevicius, Nicholas Gough, Mark Linch, Robert Grimer

**Affiliations:** ^1^Catalyst Health Economics Consultants, 34b High Street, Northwood, Middlesex HA6 1BN, UK; ^2^School of Biomedical Sciences, King's College, London SE1 1UL, UK; ^3^Palliative Care Department, Royal Marsden Hospital, London SW3 6JJ, UK; ^4^Sarcoma Unit, Royal Marsden Hospital, London SW3 6JJ, UK; ^5^Oncology Unit, Royal Orthopaedic Hospital, Birmingham B31 2AP, UK

## Abstract

Soft tissue sarcomas are a rare type of cancer generally treated with palliative chemotherapy when in the advanced stage. There is a lack of published health utility data for locally advanced “inoperable”/metastatic disease (ASTS), essential for calculating the cost-effectiveness of current and future treatments. This study estimated time trade-off (TTO) and standard gamble (SG) preference values associated with four ASTS health states (progressive disease, stable disease, partial response, complete response) among members of the general public in the UK (*n* = 207). The four health states were associated with decreases in preference values from full health. Complete response was the most preferred health state (mean utility of 0.60 using TTO). The second most preferred health state was partial response followed by stable disease (mean utilities were 0.51 and 0.43, respectively, using TTO). The least preferred health state was progressive disease (mean utility of 0.30 using TTO). The utility value for each state was significantly different from one another (*P* < 0.001). This study demonstrated and quantified the impact that different treatment responses may have on the health-related quality of life of patients with ASTS.

## 1. Introduction

Soft tissue sarcomas are malignant tumours of mesodermal cell origin accounting for 1% of all adult cancers [1]; their incidence in the United Kingdom (UK) is approximately 2,000 new cases per year [1, 2]. There are >50 different histological subtypes which vary in their clinical behaviour and response to treatment [2, 3].

Complete surgical excision, often supplemented with adjuvant radiotherapy, offers the only reliable chance of cure for localised disease [2]. However, over 50% of those having had “curative” treatment will develop metastases [4]. Although metastasectomy can be performed, palliative chemotherapy is the mainstay of treatment for locally advanced “inoperable”/metastatic disease (ASTS). The aim of such treatment is to establish disease control and improve both quantity and quality of life.

There are acknowledged clinical, histopathological, and molecular differences between soft tissue sarcoma subtypes, and to this end subtype-specific targeted therapies are being trialed [5]. However, in the UK first-line systemic chemotherapy consists of doxorubicin +/− ifosfamide for the majority of subtypes [6–8]. Second-line agents include newer cytostatic agents such as trabectedin. The prognosis for ASTS remains poor with median overall survival from commencing first- and second-line palliative chemotherapy being 10–12 and 8 months, respectively [9–11]. 

There are four levels of response following palliative chemotherapy for ASTS: progressive disease, stable disease, partial response, and complete response [12, 13]. The probability of patients with progressive disease surviving beyond 12 months is extremely low [11, 12, 14–54]. Patients with stable disease are likely to relapse within 4–9 months [12, 14, 15], and only 40–60% of patients are expected to survive beyond 16 months [11, 12, 15–30, 55–58]. Patients in partial response are likely to relapse within 5–9 months [14, 15, 31, 54, 58] and only 40–60% of patients are expected to survive beyond 18 months [11, 12, 15–30, 55–58]. Patients in complete response are likely to relapse within 10–18 months [14, 31, 54] and only 40–60% of patients are expected to survive beyond 2 years [11, 12, 15–30, 55–58].

Utilities are a measure of an individual's preference for, or desirability of, a specific level of health status or specific health outcome [59]. Quantifying the subjective impact of treatment on patients, by estimating their utility preference for different health states, is key in comparing the cost-effectiveness of alternative treatments. Estimating patients' health status utility enables preferences to be quantified for selected clinical outcomes and life expectancy to be quality adjusted. In 2010, the UK's National Institute for Health and Clinical Excellence (NICE) issued guidance on the use of trabectedin [60]. In the absence of published ASTS utilities the guidance was developed using utility values for non-small cell lung cancer as a proxy estimate for ASTS [61]. This makes the assumption that individuals' preferences for particular health status/outcomes associated with two different cancer types are identical. As such, the need to establish utility values in the ASTS population was emphasised.

Instruments used to measure an individual's health status can be generic or disease/condition specific. Moreover, the valuation of health states can be undertaken using a variety of techniques including visual analogue scales (VAS), standard gamble (SG), and time trade-off (TTO) methods. The VAS usually consists of a single line with verbal and numerical descriptors at each end. Respondents are presented with a set of health states and asked to rate their desirability for each by placing a line between the two endpoints indicative of their preference. In SG, respondents are given the choice between two alternatives: one being a health state with certainty and the other being a gamble with two possible outcomes that involve two different health states with particular probabilities attached to each of them. The probabilities attached to the health states are varied until the responder is indifferent between the two alternatives. In TTO, responders are given a choice between two health profiles: a particular health state for a given number of years and full health for a shorter period of time. In effect they are asked to trade between quality of life and length of life. The method tries to establish where they are indifferent between the two by varying the amount of time spent in full health. There is no universal agreement as to which method should be used in health economic evaluations, although it has been argued that choice-based measurement techniques such as SG and TTO are preferable to VAS [62]. Generally, TTO is widely seen as an acceptable compromise between simplicity and theoretical rigor.

The objective of this study was to estimate preference values for individual ASTS health states as they relate to the four levels of treatment response. There may be differences between the different levels of treatment response in terms of duration of response and survival, depending on whether patients were receiving first- or second-line chemotherapy. However, this study assumed that a patient's preference for each of the health states would be independent of whether they were receiving first- or second-line chemotherapy. Consequently, generalised health state descriptions were developed.

## 2. Methods

### 2.1. Health States

Descriptions of the health states under evaluation (progressive disease, stable disease, partial response, and complete response) following palliative chemotherapy were developed using published literature and clinical expert opinion [9, 11–58]. Each health state described the typical patient experience across several domains including symptoms, treatment, response, management, and prognosis, enabling a balanced description across all four health states. The health states were refined after iterative review by clinical experts and piloting the descriptions among a sample of 20 members of the general public in the UK. Full health state descriptions are listed in Table 1 and were designed to be easily understood by the general public.

### 2.2. Study Respondents

The study was undertaken among a sample of randomly selected members of the general public across the UK. This was done by randomly contacting anonymous members of the general public at six different locations across England, one location in Wales, and one in Scotland. The interviewers were unaware whether the individuals contacted had any family or friends suffering from sarcoma. Respondents had to be at least 18 years of age with or without any cancer. Potential respondents were excluded if they were non-English speaking, or if they had apparent cognitive impairment, or if in the interviewers' opinion they were incapable of understanding the task. Recruitment occurred between March and June 2012 and none of the respondents received any remuneration for participation. In order to make the sample representative, the target population was recruited according to gender distribution in different locations across the UK.

### 2.3. Sample Size

It has been suggested that a difference in utility value of 0.03 is the required minimal clinically important difference (MCID) between different health states, since this represents a difference in the risk of death of 3.5 months over a period of 10 years using TTO and 3% using SG [63]. Power calculations showed that a sample size of 185 individuals would be required to elicit a minimum difference in utility value of 0.03 between the different health states with 95% power and a type I (alpha) error of 0.05. Accordingly, this study was designed to collect data from a minimum of 185 individuals across the UK.

### 2.4. Data Collection and Analysis

Data were collected through individual, face-to-face interviews, which were conducted using an interview script. At the start of the interview the nature of the questionnaire was explained, after which participants were asked a range of sociodemographic questions about themselves. Participants were then asked to read a short, nontechnical description of ASTS and of the four different health states. They were then asked to imagine they only had two years to live and to estimate what proportion of that period they would be willing to sacrifice in return for not living with the symptoms associated with each of the four health states being evaluated.

While the aim of the study was to elicit preference values using the TTO approach, values were also elicited using the SG approach. This involved asking participants to choose between the certainty of living with the symptoms associated with each health state or gambling on a treatment with two possible outcomes: successful treatment or death. 

The search procedure used in the TTO and SG approach was simple titration and the interviewers used diagrams to help respondents visualise the trade-offs involved. No other props were used. Participants were also asked to rate their current health on a horizontal visual analogue scale (range, 0 to 1).

Utility values (ranging from 1 for perfect health to 0 for death) were obtained for the four different health states as described by Hammerschmidt et al. [64]. Differences between groups were tested for statistical significance using a Mann-Whitney *U* test. The preference values associated with the four different health states were also stratified by socio-demographic parameters including gender, age, marital status, employment status, income, and cancer status. Multiple regression was performed to assess the relationship between the aforementioned baseline parameters and outcomes.

## 3. Results

The study sample comprised 207 participants who were interviewed at one of eight locations in the UK. Overall, the participants rated their current health with a utility value of 0.83 (95% CI: 0.81; 0.86). Nine percent of participants had cancer at the time of their interview and they rated their current health with a utility value of 0.69 (95% CI: 0.59; 0.79). This was significantly lower than that of those participants who did not have cancer 0.85 (95% CI: 0.82; 0.87) (*P* < 0.01). The participants' socio-demographic details are summarised in Table 2.

Based on TTO, all four ASTS-related health states were associated with decreases in preference values from full health (Table 3). Complete response was the most preferred health state, with a mean utility of 0.60 in the overall sample. The second-most preferred health state was partial response followed by stable disease (mean utilities in the overall sample were 0.51 and 0.43, resp.). The least preferred health state was progressive disease, with a mean utility of 0.30 in the overall sample. The utility values for each state were significantly different from one another (*P* < 0.01).

The mean utility values elicited from respondents with cancer were significantly lower than the values elicited from those who did not have cancer (Table 3).

Similar trends were observed using SG (Table 3), although the utility values were all significantly lower than those derived by TTO (*P* < 0.001). Additionally, the utility values for each state were significantly different from one another (*P* < 0.001). Moreover, the differences between respondents who had and did not have cancer were smaller, particularly in the least preferred health states (i.e., progressive disease and stable disease).

Multiple regression demonstrated that respondents' preference values for any of the states were not significantly affected by age, gender, location, marital status, employment status, annual income, or whether they had cancer (Table 4). Nevertheless, respondents who were 50 years of age or younger had typically higher mean preference values than older respondents. The mean utility values elicited from respondents who were widowed were lower than those from respondents who were married/cohabiting, divorced/separated, or single (Table 4). Additionally, the mean utility values elicited from retired respondents were lower than from the other respondents. Moreover, the mean utility values elicited from those earning *£*21,000–*£*30,000 per annum were higher than from the other respondents and the values of those who were on a pension were lower (Table 4).

The mean utility values elicited from respondents using SG, stratified by their gender, age, marital status, employment status, and income are shown in Table 5. Multiple regression demonstrated that respondents' preference values for any of the states elicited using SG were affected by whether they had cancer (the utility value decreased by 0.10–0.12 among those with cancer (*P* < 0.03)) and their annual income (the utility value increased by 0.03–0.05 for every *£*10,000 of income (*P* < 0.05)). However, respondents' preference values for any of the states were not affected by their age, gender, location, marital status, and employment status.

## 4. Discussion

This study estimated TTO and SG preference values associated with four ASTS health states. The results showed that preferences for the different health states deteriorate significantly as they become more critical, from the general public's perspective. For example, the most favourable health state (complete response) evaluated by TTO was associated with a mean preference value of 0.60 compared with 0.30 for the least favourable (progressive disease). These preference values will enable a direct comparison between the different levels of treatment response to ASTS, and a more accurate calculation of quality-adjusted life years (QALYs) in economic evaluations of ASTS treatments.

In a recent cross-sectional trial among patients with metastatic soft tissue and bone sarcoma who had attained a favourable response to chemotherapy, health-related quality of life (HRQoL) was estimated using the EORTC QLQ-C30 and the EQ-5D instruments [65]. The mean EQ-5D utility score was 0.69 with little variation across health states, which was higher than preference values elicited from the general public in this study. For example, patients with ASTS valued progressive disease as 0.56, whereas the general public valued it as 0.30 using TTO. Such differences may be due to methodological differences in the way EQ-5D, TTO, and SG measure preferences for a particular health state. Additionally, the trial only recruited patients who had responded favourably to chemotherapy. The disparity between studies may also be explained by the adaptation process known as “response shift” [66] whereby patients confronted with a life-threatening condition adapt to their illness and change their “internal standards” resulting in higher evaluations of their actual health state compared to that of a noncancer patient perceiving the same health state [67].

In another study among patients with metastatic soft tissue sarcoma which assessed the cost-effectiveness of trabectedin compared with end-stage treatment after failure with anthracycline and/or ifosfamide, patients' HRQoL estimated using the QLQ-C30 scale was mapped to 15D [68], Short Form 6D, and EuroQol 5D utilities. The mean QoL index, based on the EQ-5D, was estimated to be 0.65. However, it is not clear to which health state the QoL index would be comparable to in this study. 

NICE's guidance on the use of trabectedin used utility values for non-small cell lung cancer to estimate the cost per QALY gained with this cytostatic agent [60, 61]. This guidance assumed that the utilities for progression-free and progressive-disease health states (0.65 and 0.47, resp.) would be the same for all patients, irrespective of treatment. The value of 0.65 for the progression-free health state is higher than the value for each of the progression-free health states (i.e., stable disease, partial response, and complete response) in this study. Additionally, the value of 0.47 for progressive disease in NICE's guidance was higher than the value of 0.30 for the progressive-disease health state elicited using TTO in this study. These differences show that it is potentially hazardous to assume that individuals' preferences for particular health status/outcomes associated with two different cancer types are identical. 

The mean baseline health status of our sample derived using a VAS was 0.83. Utilities were also elicited using a VAS for the four health states being studied and found to be 0.10 for progressive disease, 0.18 for stable disease, 0.24 for partial response, and 0.33 for complete response. These values were lower than the utilities elicited by TTO and SG. This is consistent with the findings of other studies, which, after a review of utilities across 995 chronic and acute health states, found a strong tendency for VAS to yield lower values than SG and TTO [69]. 

General public preference weightings remain one of the preferred options for organisations such as NICE in the UK [70] and many reimbursement agencies do not accept expert-derived utility values. However, there is no universal agreement as to which method should be used in health economic evaluations, although it has been argued that choice-based measurement techniques such as SG and TTO are preferable to visual analogue scales [62]. The utility scores obtained using SG were different from those obtained using TTO. Such differences are expected because values elicited by SG are likely to be affected by the respondents' attitudes toward risk whereas values elicited by TTO are likely to be affected by their time preferences [62]. The preferences elicited using TTO may lead to higher quality-adjusted survival calculations which would result in higher cost-effectiveness ratios for healthcare strategies when compared to using preferences elicited by SG.

Generally, TTO values are affected by respondents' time preferences and this may explain why utility values elicited from respondents tended to decrease with their increasing age. The utility values elicited from respondents with cancer in the present study were significantly lower than the values elicited from those who did not have cancer. Gender was not significantly associated with mean health state preference values and neither was location or employment status. However, respondents' time preferences were influenced by their marital status and annual income.

The strengths of this study included the use of health state descriptions developed through literature review and clinical expert opinion, which were pilot-tested to ensure understanding by the respondents and validity of the resultant ASTS health state preference values. In order to make the sample representative, the target population was recruited according to gender distribution in eight different locations across the UK, although participants were not recruited according to age. Notwithstanding this, the possibility that the respondents may not have been broadly representative of the target population of the UK as a whole cannot be excluded. NICE [70] and other agencies, such as the Scottish Medicines Consortium (SMC) [71], accept the use of general public-based utilities. However, NICE has a preference for utilities derived from using the EQ-5D classification system. If such values are not available, NICE has a preference for utilities elicited using a TTO among community-based respondents. Other agencies in the UK (such as the SMC) are less prescriptive and accept utilities derived from a variety of methods (such as TTO or SG), as long as the values generated for the health states appear plausible. Undoubtedly, validating utility estimates for each health state among a population of patients with ASTS in the future may be advantageous, since this study suggests that cancer sufferers had less preference for each health state than members of the general public who did not have cancer.

This study was subject to a number of other limitations. Firstly, interviews with ASTS patients were not included as part of the health state development process for practical reasons, although feedback from patients would have facilitated validation of the health state descriptions. Instead, the study relied on contributions from clinical experts who treat ASTS and piloting among members of the general public to validate the health states descriptions. Secondly, as required by the TTO approach, it was assumed that the relationship between the duration of living in a health state and an individual's utility value for that health state was independent. This assumption may not be valid since a study using EQ-5D health states found that preferences decline with increasing duration of remaining in a severe health state [72]. Further studies are required to assess whether this assumption is valid for health state valuations in cancer using a TTO approach and whether the elicited utility values overestimate true preferences for ASTS health states. Thirdly, it was assumed that a patient's preference for each of the health states would be independent of whether they were receiving first- or second-line chemotherapy. This assumption may not be valid and further studies are required to assess whether a patient's preference for an individual health state is affected by the stage of their cancer.

In conclusion, this study indicates greater preference values for enhanced levels of treatment response and demonstrates the impact that different treatment responses may have on the HRQoL of patients with ASTS, from the general public's perspective. These health state preference values can be used to estimate the outcomes of interventions in terms of QALYs.

## Figures and Tables

**Table 1:** Health state descriptions for the four ASTS health states.

ASTS	
(i) ASTS is an incurable cancer of no known cause. It often originates in the limbs or trunk and commonly spreads to the lungs, lymph nodes, and bones.	
(ii) ASTS frequently presents with a painful swelling or lump. However, symptoms depend on which part of the body is affected and may include pain, cough, breathlessness, nausea, vomiting, constipation, and fatigue.	
(iii) Family practitioners would refer patients to hospital for specialist tests that may include X-rays, ultrasound scans, MRI/CT scans, and a biopsy to make the diagnosis.	
(iv) Treatment largely involves chemotherapy and/or radiotherapy with the intention of controlling the disease and improving symptoms.	
(v) Chemotherapy is generally administered every 21–28 days as an outpatient or inpatient. During treatment, patients are at risk of infections which may result in more doctor visits and hospital admissions. There are four outcomes to treatment: complete response, partial response, stable disease, and progressive disease.	

Progressive disease	
(i) Patients with progressive disease have not responded to chemotherapy. They may receive further chemotherapy or palliative care and frequently experience weight loss, nausea/vomiting, breathlessness, cough, constipation, and fatigue.	
(ii) They may need help with day-to-day activities, for example, washing/dressing, and are likely to experience pain needing strong pain killers.	
(iii) The chance of surviving beyond 12 months is rare and patients often experience higher levels of anxiety/depression for which they may receive medication.	

Stable disease	
(i) Patients with stable disease will almost certainly relapse within 4–9 months. Their cancer is no longer progressing, but they continue to experience symptoms.	
(ii) During treatment they may lose weight and have nausea/vomiting, constipation, and fatigue.	
(iii) They may require help performing day-to-day activities and be in some pain for which they require pain killers.	
(iv) The chance of surviving beyond 16 months is 40–60% and patients are also likely to be anxious and depressed for which they may receive medication.	

Partial response	
(i) Patients in partial response will almost certainly relapse within 5–9 months.	
(ii) After treatment, patients will have an improvement in pain, other symptoms, and level of activity, but less so than those with complete response.	
(iii) During treatment patients may lose weight and have nausea/vomiting, constipation, and fatigue.	
(iv) The chance of surviving beyond 18 months is 40–60%.	

Complete response	
(i) Patients in complete response will almost certainly relapse within 10–18 months.	
(ii) After treatment, patients are likely to have minimal pain and few symptoms and be active.	
(iii) During treatment they may lose weight and have nausea/vomiting, constipation, and fatigue.	
(iv) The chance of surviving beyond 2 years is 40–60%.	

**Table 2:** Respondents' socio-demographic characteristics.

Characteristic	Mean number (with 95% confidence intervals) or percent
Respondent's age	54.8 (52.7; 57.0) years
Percent female	53%
Marital status	
Percent married/cohabiting	61%
Percent single	21%
Percent divorced/separated	9%
Percent widowed	9%
Employment status	
Percent employed	59%
Percent retired	35%
Percent students	1%
Percent unemployed	4%
Percent at home	1%
Mean annual income	*£*22,900 (*£*21,300; *£*24,500)
Percent with cancer at the time of the interview	9% had cancer for a mean 0.8 (0.1; 1.7) years
Percent of respondents who knew individuals with cancer	66%

**Table 3:** Mean utilities (95% confidence intervals) for ASTS health states, stratified by respondents' cancer status and elicitation method.

	Mean utilities (95% confidence intervals) for			
	Progressive disease	Stable disease	Partial response	Complete response
Utilities elicited using TTO				
Whole cohort	0.30 (0.26; 0.34)	0.43 (0.39; 0.47)	0.51 (0.47; 0.55)	0.60 (0.57; 0.64)
Respondents with cancer	0.16 (0.06; 0.27)*	0.26 (0.17; 0.35)**	0.34 (0.25; 0.44)***	0.48 (0.36; 0.60)****
Respondents without cancer	0.31 (0.26; 0.35)*	0.44 (0.40; 0.49)**	0.53 (0.48; 0.57)***	0.61 (0.58; 0.65)****
Utilities elicited using SG				
Whole cohort	0.17 (0.14; 0.19)	0.31 (0.29; 0.34)	0.43 (0.40; 0.45)	0.51 (0.49; 0.53)
Respondents with cancer	0.07 (0.01; 0.12)^†^	0.21 (0.15; 0.28)^††^	0.32 (0.25; 0.39)^†††^	0.42 (0.34; 0.50)^††††^
Respondents without cancer	0.18 (0.14; 0.21)^†^	0.32 (0.30; 0.35)^††^	0.44 (0.41; 0.46)^†††^	0.52 (0.49; 0.54)^††††^

*P < 0.05; **P < 0.02; ***P = 0.01; ****P = 0.05; ^†^ *P* < 0.03; ^††^ *P* < 0.02; ^†††^ *P* < 0.01; ^††††^ *P* < 0.05.

**Table 4:** Mean utilities (95% confidence intervals) for ASTS health states, stratified by socio-demographic parameters, using TTO. (*15 respondents refused to provide details about their income).

	Mean utilities (95% confidence intervals) for			
	Progressive disease	Stable disease	Partial response	Complete response
Respondents' gender				
Male (*n* = 98)	0.26 (0.20; 0.32)	0.40 (0.34; 0.46)	0.49 (0.44; 0.55)	0.59 (0.54; 0.63)
Female (*n* = 109)	0.33 (0.27; 0.38)	0.45 (0.39; 0.51)	0.53 (0.48; 0.59)	0.62 (0.57; 0.66)
Respondents' age				
<41 years (*n* = 60)	0.32 (0.24; 0.41)	0.47 (0.38; 0.56)	0.53 (0.44; 0.62)	0.62 (0.55; 0.69)
41–50 years (*n* = 28)	0.45 (0.35; 0.55)*	0.55 (0.45; 0.65)**	0.63 (0.53; 0.72)***	0.71 (0.63; 0.79)****
51–60 years (*n* = 42)	0.23 (0.13; 0.33)	0.40 (0.30; 0.51)	0.51 (0.42; 0.60)	0.58 (0.51; 0.65)
61–70 years (*n* = 37)	0.27 (0.17; 0.36)	0.37 (0.27; 0.46)	0.46 (0.36; 0.56)	0.57 (0.49; 0.64)
>70 years (*n* = 40)	0.24 (0.17; 0.31)	0.37 (0.30; 0.43)	0.46 (0.39; 0.54)	0.57 (0.51; 0.63)
Respondents' marital status				
Single (*n* = 44)	0.35 (0.24; 0.45)	0.54 (0.44; 0.64)	0.61 (0.52; 0.71)	0.67 (0.58; 0.75)
Married/cohabiting (*n* = 127)	0.31 (0.27; 0.36)	0.43 (0.38; 0.48)	0.52 (0.47; 0.57)	0.62 (0.58; 0.65)
Divorced/separated (*n* = 18)	0.25 (0.09; 0.40)	0.42 (0.29; 0.55)	0.48 (0.36; 0.60)	0.58 (0.47; 0.69)
Widowed (*n* = 18)	0.09 (0.02; 0.16)^†^	0.20 (0.08; 0.33)^††^	0.28 (0.14; 0.41)^†††^	0.41 (0.30; 0.52)^††††^
Respondents' employment status				
Employed (*n* = 110)	0.31 (0.25; 0.37)	0.45 (0.39; 0.51)	0.53 (0.47; 0.58)	0.61 (0.57; 0.66)
Self-employed (*n* = 11)	0.32 (0.14; 0.51)	0.47 (0.26; 0.68)	0.55 (0.36; 0.75)	0.67 (0.54; 0.81)
Unemployed (*n* = 8)	0.25 (0.00; 0.54)	0.42 (0.30; 0.55)	0.55 (0.47; 0.63)	0.68 (0.49; 0.87)
Retired (*n* = 72)	0.25 (0.19; 0.31)	0.36 (0.30; 0.42)	0.46 (0.40; 0.53)	0.56 (0.50; 0.61)
Student/at home (*n* = 6)	0.55 (0.38; 0.72)	0.60 (0.43; 0.77)	0.67 (0.49; 0.84)	0.72 (0.51; 0.94)
Respondents' income*				
Pension (*n* = 43)	0.24 (0.18; 0.31)^‡^	0.35 (0.29; 0.42)^‡‡^	0.43 (0.37; 0.50)^‡‡‡^	0.55 (0.49; 0.60)
<*£*10,000 (*n* = 17)	0.26 (0.14; 0.37)	0.40 (0.29; 0.50)	0.51 (0.39; 0.63)	0.61 (0.51; 0.71)
*£*10,000–*£*20,000 (*n* = 34)	0.30 (0.20; 0.40)	0.41 (0.34; 0.48)	0.50 (0.42; 0.58)	0.63 (0.56; 0.70)
*£*21,000–*£*30,000 (*n* = 37)	0.37 (0.27; 0.47)	0.52 (0.41; 0.63)	0.59 (0.50; 0.69)	0.64 (0.56; 0.72)
>*£*30,000 (*n* = 61)	0.31 (0.21; 0.40)	0.47 (0.37; 0.57)	0.55 (0.46; 0.64)	0.62 (0.55; 0.69)

*P < 0.02; **P < 0.03; ***P < 0.05; ****P < 0.01 higher than the three older age groups.

^†^ *P* < 0.002; ^††^ *P* < 0.002; ^†††^ *P* < 0.005; ^††††^ *P* < 0.001 lower than the single and married/cohabiting groups.

^‡^ *P* < 0.05; ^‡‡^ *P* < 0.01; ^‡‡‡^ *P* < 0.02; lower than the *£*21,000–*£*30,000 group.

**Table 5:** Mean utilities (95% confidence intervals) for ASTS health states, stratified by socio-demographic parameters, using SG. (*15 respondents refused to provide details about their income).

	Mean utilities (95% confidence intervals) for			
	Progressive disease	Stable disease	Partial response	Complete response
Respondents' gender				
Male (*n* = 98)	0.17 (0.13; 0.21)	0.31 (0.27; 0.35)	0.44 (0.40; 0.47)	0.51 (0.48; 0.55)
Female (*n* = 109)	0.16 (0.12; 0.20)	0.32 (0.28; 0.35)	0.42 (0.39; 0.45)	0.51 (0.47; 0.54)
Respondents' age				
<41 years (*n* = 60)	0.15 (0.10; 0.20)	0.32 (0.28; 0.36)	0.43 (0.39; 0.47)	0.51 (0.47; 0.55)
41–50 years (*n* = 28)	0.16 (0.09; 0.23)	0.32 (0.26; 0.38)	0.44 (0.39; 0.50)	0.53 (0.47; 0.58)
51–60 years (*n* = 42)	0.18 (0.12; 0.24)	0.34 (0.29; 0.38)	0.45 (0.40; 0.50)	0.54 (0.49; 0.59)
61–70 years (*n* = 37)	0.16 (0.09; 0.23)	0.28 (0.21; 0.34)	0.39 (0.33; 0.46)	0.48 (0.42; 0.53)
>70 years (*n* = 40)	0.19 (0.10; 0.27)	0.31 (0.24; 0.39)	0.43 (0.36; 0.49)	0.50 (0.43; 0.56)
Respondents' marital status				
Single (*n* = 44)	0.19 (0.12; 0.26)	0.32 (0.27; 0.38)	0.42 (0.36; 0.48)	0.51 (0.45; 0.56)
Married/cohabiting (*n* = 127)	0.16 (0.12; 0.19)	0.31 (0.28; 0.34)	0.44 (0.41; 0.47)	0.52 (0.49; 0.55)
Divorced/separated (*n* = 18)	0.15 (0.07; 0.23)	0.31 (0.24; 0.39)	0.39 (0.31; 0.47)	0.46 (0.39; 0.53)
Widowed (*n* = 18)	0.18 (0.05; 0.30)	0.31 (0.19; 0.43)	0.41 (0.30; 0.51)	0.49 (0.39; 0.59)
Respondents' employment status				
Employed (*n* = 110)	0.14 (0.10; 0.17)	0.31 (0.28; 0.34)	0.43 (0.40; 0.46)	0.52 (0.49; 0.55)
Self-employed (*n* = 11)	0.11 (0.03; 0.19)	0.29 (0.21; 0.36)	0.38 (0.29; 0.47)	0.48 (0.38; 0.57)
Unemployed (*n* = 8)	0.26 (0.05; 0.48)	0.30 (0.11; 0.49)	0.40 (0.26; 0.54)	0.46 (0.33; 0.60)
Retired (*n* = 72)	0.21 (0.15; 0.27)	0.32 (0.27; 0.37)	0.43 (0.38; 0.48)	0.50 (0.45; 0.55)
Student/at home (*n* = 6)	0.14 (0.00; 0.29)	0.34 (0.15; 0.53)	0.41 (0.20; 0.62)	0.50 (0.37; 0.63)
Respondents' income*				
Pension (*n* = 43)	0.06 (0.02; 0.10)	0.22 (0.17; 0.27)	0.34 (0.29; 0.39)	0.42 (0.37; 0.47)
<*£*10,000 (*n* = 17)	0.23 (0.10; 0.36)	0.35 (0.23; 0.46)	0.44 (0.34; 0.54)	0.55 (0.46; 0.64)
*£*10,000–*£*20,000 (*n* = 34)	0.23 (0.15; 0.31)	0.38 (0.32; 0.45)	0.49 (0.43; 0.55)	0.55 (0.49; 0.61)
*£*21,000–*£*30,000 (*n* = 37)	0.19 (0.12; 0.26)	0.33 (0.28; 0.38)	0.46 (0.40; 0.52)	0.54 (0.48; 0.60)
>*£*30,000(*n* = 61)	0.14 (0.10; 0.18)	0.31 (0.27; 0.34)	0.43 (0.40; 0.46)	0.53 (0.49; 0.56)
